# Die Zertifizierung von Zentren für Beatmungsentwöhnung in der neurologisch-neurochirurgischen Frührehabilitation durch die Deutsche Gesellschaft für Neurorehabilitation

**DOI:** 10.1007/s00115-021-01207-9

**Published:** 2021-10-14

**Authors:** Martin Groß, Marcus Pohl, Thomas Platz, Tobias Schmidt-Wilcke

**Affiliations:** 1 Zertifizierungsausschuss der Deutschen Gesellschaft für Neurorehabilitation e. V., Rheinbach, Deutschland; 2 Oldenburger Forschungsnetzwerk Notfall- und Intensivmedizin, Carl von Ossietzky Universität Oldenburg, Oldenburg, Deutschland; 3 Evangelisches Krankenhaus Oldenburg, Oldenburg, Deutschland; 4 VAMED Klinik Schloss Pulsnitz, Pulsnitz, Deutschland; 5 Präsidium der Deutschen Gesellschaft für Neurorehabilitation e. V., Rheinbach, Deutschland; 6 Institut für Neurorehabilitation und Evidenzbasierung, An-Institut der Universität Greifswald, BDH-Klinik Greifswald gGmbH, Zentrum für NeuroRehabilitation Beatmungs- und Intensivmedizin Querschnittgelähmtenzentrum, Karl-Liebknecht-Ring 26a, 17491 Greifswald, Deutschland; 7 Arbeitsgruppe Neurorehabilitation, Universitätsmedizin Greifswald, Greifswald, Deutschland; 8 Neurologisches Zentrum, Mainkofen, Deutschland; 9 Institut für Klinische Neurowissenschaften und Medizinische Psychologie, Heinrich-Heine – Universität Düsseldorf, Düsseldorf, Deutschland

**Keywords:** Beatmungsentwöhnung, Deutsche Gesellschaft für Neurorehabilitation, Zertifizierung, Neurologisch-neurochirurgische Frührehabilitation, Intensivpflege, Ventilation weaning, German Society for Neurorehabilitation, Certification, Neurological neurosurgical early rehabilitation, Intensive care

## Abstract

Die Zertifizierung von Zentren für Beatmungsentwöhnung in der neurologisch-neurochirurgischen Frührehabilitation durch die Deutsche Gesellschaft für Neurorehabilitation (DGNR) ist ab dem 01.10.2021 möglich. Die Zertifizierungskriterien beschreiben ein Anforderungsprofil, das für eine fachgerechte und qualitätsgesicherte Versorgung von Beatmungspatienten in der neurologisch-neurochirurgischen Frührehabilitation (NNFR) steht. Das Zertifikat berücksichtigt die strukturellen Unterschiede der in der NNFR tätigen Einrichtungen und kann sowohl durch Facheinrichtungen als auch durch Frührehabilitationsabteilungen an Akutkrankenhäusern erworben werden. Die Durchführung der Zertifizierung erfolgt analog zur Zertifizierung von Stroke-Units der Deutschen Schlaganfall-Gesellschaft in Zusammenarbeit mit dem TÜV Rheinland. Zunächst sendet die Einrichtung den Erhebungsbogen an den TÜV Rheinland. Anschließend erfolgt die Begehung durch einen vom TÜV Rheinland gestellten leitenden Auditor und einen Fachauditor der DGNR. Deren Bericht wird dem Zertifizierungsausschuss der DGNR vorgelegt zur Erteilung einer Empfehlung oder Ablehnung der Zertifizierung. Die Zertifizierung schafft objektive Kriterien, die die Rolle der Neurologie in der Beatmungsmedizin in Deutschland beschreiben. So erleichtert sie den Dialog mit anderen beatmungsmedizinischen Disziplinen und ebnet den Weg für die Diskussion mit Politikern, Kostenträgern und nicht zuletzt Betroffenenverbänden über Behandlungsinhalte und -kapazitäten.

## Abstract (en)

Specialized centers for weaning in neurological and neurosurgical early rehabilitation can be certified by the German Society for Neurorehabilitation (DGNR) since 1 October 2021. The certification criteria are indicators for structure and process quality and for treatment results for weaning of neurological patients from mechanical ventilation. In Germany these patients are treated in departments for neurological and neurosurgical early rehabilitation in acute care hospitals as well as in specialized rehabilitation hospitals. Acknowledging this diversity of care, certification is possible for both kinds of institutions. Certification is granted by the DGNR in cooperation with TÜV Rheinland as the certification authority, analogous to the certification of stroke units. Institutions apply for certification by sending all necessary documents to the TÜV Rheinland. Afterwards auditors of the TÜV Rheinland (lead auditor) and the DGNR (medical speciality auditor) visit the institution on site. The results of this visit are reported to the certification committee of the DGNR, which in turn recommends or declines certification. The certification of centers for weaning in neurological and neurosurgical early rehabilitation is an important step towards defining neurorespiratory care and the role of the neurologist for intensive care and weaning in Germany. Thereby, the interdisciplinary dialogue between pneumologists, anesthesiologists, and neurologists is fostered. Also, the dialogue on treatment capacities and strategies with healthcare politicians, health insurances, and patient associations is facilitated.

## Aktuelle Rahmenbedingungen

Deutschland verfügt über die höchste Zahl an Intensivbetten im Verhältnis zu seiner Bevölkerungszahl in Europa [1]. Eine Erhebung der Deutschen Gesellschaft für Neurorehabilitation (DGNR) zeigte, dass es in Deutschland ca. 1100 Beatmungsbetten im Rahmen der neurologisch-neurochirurgischen Frührehabilitation (NNFR) gibt [2]. Des Weiteren werden in Deutschland 26.000 Patienten der außerklinischen Intensivpflege (AKI) in der Häuslichkeit, in Intensivpflege-WGs oder stationären Intensivpflegeeinrichtungen versorgt [3]. Neurologische Patienten sind mit > 50 % die bedeutendste Klientel der außerklinischen Intensivpflege [4]. Auf Beatmungsentwöhnung sowie NNFR spezialisierte Einrichtungen sind an einer wichtigen Schnittstelle tätig: Sie können die Beatmungs- und Trachealkanülenentwöhnung von aus der Akutversorgung übernommenen Patienten durchführen und dadurch nicht selten die Notwendigkeit der AKI vermeiden. Wenn dies nicht möglich ist, werden trotzdem die Voraussetzungen für eine möglichst hohe Lebensqualität, gleichberechtigte Teilhabe und medizinische Stabilität geschaffen, bevor der Patient in die AKI entlassen wird. Zudem verfügen auf Beatmungsentwöhnung und NNFR spezialisierte Einrichtungen über die erforderliche Expertise, um Patienten aus der außerklinischen Intensivpflege zur Beatmungs- und Trachealkanülenentwöhnung aufzunehmen.

Die Entwöhnung von der Beatmung und Dekanülierung führen neben einer Verbesserung der Lebensqualität zu einer ökonomischen Entlastung der Kostenträger, da nach gelungener Dekanülierung in der Regel keine AKI mehr erforderlich ist. Die Kosten der AKI werden mit bis zu 4 Mrd. € pro Jahr geschätzt. Es gibt Hinwiese, dass in Deutschland die Bettenkapazitäten für die Beatmungsentwöhnung derzeit nicht ausreichen: 85 % der Patienten der außerklinischen Intensivpflege werden ohne vorherige Behandlung in einem spezialisierten Zentrum zuverlegt [5], und in der Region Niedersachsen-Bremen erhielten nur 45 % aller für die neurologisch-neurochirurgische Frührehabilitation angemeldeten Patienten insgesamt und lediglich 37 % der beatmeten Patienten einen Behandlungsplatz [6]. Es kann somit davon ausgegangen werden, dass bei einem relevanten Anteil der Patienten in der AKI die Beatmungsentwöhnung und sogar die Dekanülierung möglich sind. Der Ausbau der NNFR mit und ohne Beatmungsentwöhnung ist somit sozioökonomisch hoch relevant.

Von Seiten des Gesetzgebers wurde die Notwendigkeit der Beatmungsentwöhnung in spezialisierten Einrichtungen erkannt und in das 2020 verabschiedete Intensivpflege- und Rehabilitationsstärkungsgesetz (IPREG) eingebracht. Zukünftig werden Krankenhäuser, die die Beatmungsentwöhnungspotenziale der Patienten nicht ausschöpfen, Patienten also nicht in spezialisierte Zentren verlegen, mit finanziellen Abschlägen rechnen müssen. Die Regelung bezweckt, dass mehr Betroffene erfolgreich von der Beatmung und Trachealkanüle entwöhnt werden. Wenn aber Kapazitäten in spezialisierten Zentren fehlen, ist allerdings aus Sicht der Patienten, aber auch der Kostenträger vom IPREG kein einschlägiger Effekt zu erwarten. Eine bedarfsgerechte Vorhaltung (und ggf. Aufbau) von Betten in den Zentren für Beatmungsentwöhnung in der NNFR ist daher erforderlich. Die DGNR möchte mit ihrem Zertifizierungsverfahren einen Qualitätsstandard etablieren, der für eine fachgerechte und qualitätsgesicherte Versorgung in der Beatmungsentwöhnung neurologisch Erkrankter steht.

## Bedeutung der Zertifizierung in Bezug auf die Abbildung der Beatmungsentwöhnung im Operationen- und Prozedurenschlüssel

Mittlerweile ist die Beatmungsentwöhnung im Operationen- und Prozedurenschlüssel (OPS, Version 2021) durch die Ziffern 8‑718.7 (Beatmungsentwöhnung nicht auf Beatmungsentwöhnungs-Einheit), 8‑718.8 (Prolongierte Beatmungsentwöhnung auf spezialisierter intensivmedizinischer Beatmungsentwöhnungs-Einheit) und 8‑718.9 (Prolongierte Beatmungsentwöhnung auf spezialisierter nicht intensivmedizinischer Beatmungsentwöhnungs-Einheit) abgebildet. Somit ist die Spezialisierung im Bereich der Beatmungsentwöhnung erlösrelevant geworden. „Krankenhäuser haben die Einhaltung von Strukturmerkmalen aufgrund des vom Bundesinstitut für Arzneimittel und Medizinprodukte herausgegebenen Operationen- und Prozedurenschlüssels nach § 301 Absatz 2 durch den Medizinischen Dienst (MD) begutachten zu lassen, bevor sie entsprechende Leistungen abrechnen“ (SGB 5, § 275d, Abs. 1, Satz 1). Das exakte Vorgehen des MD bei der Prüfung der Strukturmerkmale der die Beatmungsentwöhnung betreffenden OPS ist noch nicht bekannt.

Die für die OPS 8‑718.8 und 8-718.9 und für die Zertifizierung geforderten Strukturmerkmale sind grundsätzlich vergleichbar, unterscheiden sich aktuell aber in wichtigen Details: In der Strukturprüfung des MD werden derzeit eine 24-stündige Verfügbarkeit der Bronchoskopie, das Vorhalten mechanischer Insufflatoren-Exsufflatoren und die Möglichkeit zum ethischen Fallgespräch gefordert. All dies wird für die Zertifizierung durch die DGNR zwar erwartet, das Nichtvorhandensein schließt zum jetzigen Zeitpunkt die Zertifizierung jedoch nicht aus. Die Zertifizierung durch die DGNR setzt wiederum Expertise in der fiberendoskopischen Untersuchung des Schluckaktes, nachzuweisen durch ein FEES-Zertifkat, und eine Mindestanzahl von 40 Beatmungsentwöhnungsbehandlungen pro Jahr voraus. Eingereicht werden müssen für die Zertifizierung durch die DGNR schon bei der Beantragung Konzepte für das Atemwegsmanagement, die Beatmungsentwöhnung, das Trachealkanülenmanagement und das Dysphagiemanagement. Für die OPS 8‑718.8 und 8-718.9 wird lediglich die Darstellung einer auf Beatmungsentwöhnung spezialisierten Einheit gefordert, was z. B. in Form eines Konzeptes/einer „standardised operation procedure“ (SOP) erfolgen kann.

Die Zertifizierung durch die DGNR prüft zudem nicht nur Struktur-, sondern auch Prozessmerkmale inklusive Mindestmerkmale mit dem Anspruch, die Beatmungsentwöhnung in die NNFR einzubetten. Erheblich unterscheiden sich die in den OPS 8‑718.8 und 8-718.9 von den in der OPS 8‑552 (NNFR) sowie der Zertifizierung geforderten Therapieminuten – 300 min pro Woche gegenüber 300 min pro Tag. Die Einhaltung der Therapieminuten stellt allerdings für die Zertifizierung ein Mindestmerkmal im Bereich der Prozessqualität dar.

## Beatmungsentwöhnung in der neurologisch-neurochirurgischen Frührehabilitation

Die Beatmungsentwöhnung bei neurologischen Patienten stellt eine besondere Herausforderung dar. Ursächlich hierfür sind unter anderem demographisch bedingte Veränderungen der Altersstruktur von Intensivpatienten [7]. Mit höherem Lebensalter steigt das Risiko, an einer neurologischen Erkrankung oder an einer neurologischen Komplikation einer – meist internistischen oder chirurgischen – Primärerkrankung (z. B. Critical-illness-Polyneuropathie/-Myopathie) so schwer zu erkranken, dass eine Beatmung auf einer Intensivstation sowie eine anschließende NNFR mit Beatmungs- und Trachealkanülenentwöhnung notwendig werden.

Innerhalb der Fachbereiche Pneumologie, Anästhesiologie und Neurologie sind jeweils spezialisierte Versorgungsstrukturen entstanden, die Beatmungsentwöhnung durchführen. Im neurologischen Fachbereich findet diese vor allem im Rahmen der NNFR statt [8, 9]. In der NNFR findet sowohl während der Beatmungsentwöhnung als auch danach eine an die Belastbarkeit des Patienten adaptierte Neurorehabilitation statt, mit dem Ziel, den Patienten während des gesamten Behandlungsverlaufs optimal zu fördern. Die Behandlung beatmeter Patienten findet in der NNFR in folgenden Organisationsformen statt:

- Auf Frührehabilitation spezialisierte Einrichtungen (Fachkrankenhäuser, Frührehabilitationseinrichtungen), welche häufig zunächst über einen Frührehabilitationsbereich verfügten und an denen später ein Intensiv‑/Beatmungsbereich aufgebaut wurde. Vorteile dieser Organisationsform sind, dass die Phasen der Neurorehabilitation kontinuierlich durchlaufen werden können („integrierte Neurorehabilitation“ [10]) und meist umfangreiche Erfahrung in der Neurorehabilitation und zeitgemäße Rehabilitationstechnologie vorhanden sind.
- Akutkrankenhäuser, die häufig zunächst nicht über eine NNFR verfügten und an denen diese erst später aufgebaut wurde. Vorteile dieser Organisationsform sind in der Regel vorhandene umfangreiche Erfahrung in der Intensivmedizin und die Möglichkeit, auch schwere medizinische Komplikationen vor Ort behandeln zu können.

In der NNFR existierte bisher noch keine Zertifizierung für auf Beatmungsentwöhnung spezialisierte Einheiten anhand einheitlicher Kriterien für die Struktur- und Prozessqualität. Die Zertifizierung von Zentren für Beatmungsentwöhnung in der neurologisch-neurochirurgischen Frührehabilitation erfolgt nun anhand eines Anforderungsprofils, dessen Erfüllung für eine fachgerechte und qualitätsgesicherte Versorgung von Beatmungspatienten in der NNFR steht (Formblatt abrufbar unter https://www.dgnr.de/zertifizierung). Der Aufbau des Zertifikats berücksichtigt die strukturellen Unterschiede der in der DGNR vertretenen Einrichtungen und ermöglicht sowohl Facheinrichtungen als auch Frührehabilitationsabteilungen an Akutkrankenhäusern den Erwerb des Zertifikats. Die Zertifizierung schafft objektive Kriterien, die die Rolle der Neurologie in der Beatmungsmedizin in Deutschland beschreiben und gegenüber anderen Fachgesellschaften, Kostenträgern und letztendlich der Politik kommuniziert werden können, was die Diskussion über Behandlungsinhalte, Behandlungskapazitäten und Erlöse vereinfachen wird.

## Auf Beatmungsentwöhnung und außerklinische Beatmung spezialisierte Einrichtungen in Deutschland

Die Deutsche Gesellschaft für Pneumologie und Beatmungsmedizin e. V. (DGP) führt seit über 10 Jahren anhand definierter Struktur- und Prozesskriterien eine Zertifizierung pneumologisch orientierter Weaningzentren durch [11]. Neben der Beachtung bestimmter Strukturen und Prozessmerkmale spielt die Eingabe von Daten in eine zentrale Datenbank eine wichtige Rolle [12]. Etwa 60 Weaningzentren waren im September 2021 zertifiziert [13].

Von der Deutschen Gesellschaft für Anästhesiologie und Intensivmedizin (DGAI) wurde im Rahmen des modularen Zertifikats „Intensivmedizin“ das Zertifikat „Entwöhnung von der Beatmung“ eingeführt [14]. Die Zahl der zertifizierten Einheiten wurde bisher nicht veröffentlicht.

Die Deutsche Interdisziplinäre Gesellschaft für Außerklinische Beatmung (DIGAB) plant die Zertifizierung von Zentren für außerklinische Beatmung. Da ca. 8 % der Patienten der neurologischen Beatmungsentwöhnungseinheiten beatmet in die außerklinische Versorgung entlassen werden [8], ergibt sich eine inhaltliche Schnittstelle der Zentren für Beatmungsentwöhnung in der NNFR mit den Zentren für außerklinische Beatmung. Eine Teilnahme der Zentren für Beatmungsentwöhnung in der NNFR an dieser Versorgungsform – z. B. mit Ermächtigungsambulanzen, Medizinische Zentren für Erwachsene mit Behinderung (MZEB), Tageskliniken oder Telemedizinangeboten – kann die Versorgung außerklinisch intensivpflichtiger Patienten erheblich verbessern.

Des Weiteren sind an der Versorgung beatmeter Patienten in Deutschland Zentren für Querschnittgelähmtenbehandlung beteiligt. Diese verfügen über Expertise in der Beatmungsentwöhnung, (Früh-)Rehabilitation, Hilfsmittelversorgung und außerklinischen Beatmung bei querschnittgelähmten Patienten. Zudem liegt in einigen dieser Zentren Expertise in der Versorgung mit Zwerchfellschrittmachern vor. Eine grundsätzliche Zertifizierung der Querschnittgelähmtenzentren erfolgt durch die Deutschsprachige Medizinische Gesellschaft für Paraplegiologie (DMGP).

In Schlaflaboren, die von der Deutschen Gesellschaft für Schlafforschung und Schlafmedizin (DGSM) zertifiziert werden, wird die nichtinvasive Beatmung bei nicht intensivpflichtigen Patienten eingestellt und kontrolliert. Das Behandlungsspektrum umfasst Schlafapnoesyndrome, COPD, das Obesitas-Hypoventilationssyndrom, Kyphoskoliosen und neuromuskuläre Erkrankungen.

**Figure 1:**
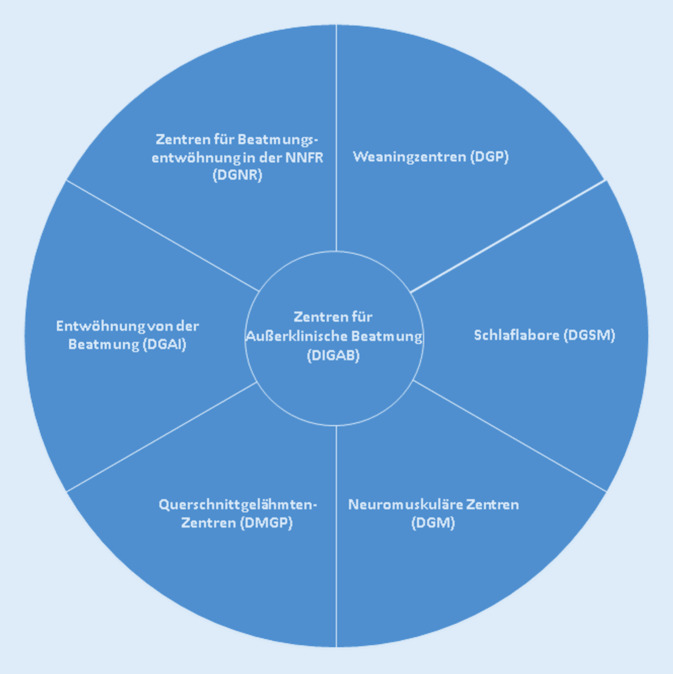

Schließlich existieren auch an einigen neuromuskulären Zentren beatmungsmedizinische Konzepte, die Beatmungseinstellung, die Beatmungskontrolle und die Notfallbehandlung bei neuromuskulären Patienten umfassen. Beatmung findet dort auch auf Normalstationen statt, da diese bei gegebener Expertise des Personals für Patienten mit neuromuskulären Erkrankungen als geeigneter eingeschätzt werden als Intensivstationen. Die Zertifizierung erfolgt durch die Deutsche Gesellschaft für Muskelkranke (DGM). Abb. 1 gibt einen Überblick über die Versorgungsstrukturen in der Beatmungsmedizin in Deutschland.

## Besondere Herausforderungen der Beatmungsentwöhnung in der Neurologie

Die in der Beatmungsentwöhnung tätigen medizinischen Fachbereiche haben unterschiedliche Expertisen. Die Beatmungsentwöhnung im Fachbereich der Neurologie findet in aller Regel im konzeptionellen und organisatorischen Rahmen der NNFR statt, die auf Patienten mit neurologischen Erkrankungen ausgerichtet ist. In der NNFR wird die Beatmungsentwöhnung als erster Teil der Neurorehabilitation verstanden und gelebt, und im Durchschnitt werden täglich mindestens 300 Therapieminuten verordnet. Nur so können die oft komplexen Erkrankungsfolgen – z. B. in den Bereichen Bewusstsein, Kognition, Kommunikation, Sensorik, Motorik – insgesamt adäquat behandelt werden.

Mittlerweile wird auch eine zunehmende Zahl schwer betroffener Patienten mit einer initial nichtneurologischen Erkrankung und einer komplizierenden Critical-illness-Poly- und -Myopathie im Rahmen des „post intensive care syndrome“ (PICS) in der NNFR behandelt. Dieser Umstand trägt dazu bei, dass das Behandlungsspektrum in der NNFR zunehmend die Intensivmedizin, internistische und chirurgische Komorbiditäten umfasst.

Die Beatmungsentwöhnung wird bei neurologischen Patienten häufig durch Störungen des Atemantriebs, der Atempumpe, des Schluckens und des Hustens sowie durch eine tracheobronchiale Sekretretention und Aspirationspneumonien kompliziert. Voraussetzungen der erfolgreichen Beatmungs- und Trachealkanülenentwöhnung und sind die Vermeidung von Aspirationspneumonien, ein suffizienter Hustenstoß und das Erlernen eines Schluckakts, der ein ausreichendes Speichelmanagement ermöglicht und einen Schutz vor Aspiration bietet. Dieser Lernprozess setzt neben einer ausreichenden pharyngealen und laryngealen Sensibilität ein gewisses Maß an Kooperationsfähigkeit voraus, welche häufig erst durch begleitende multiprofessionelle Frührehabilitation inkl. Neuropsychologie erarbeitet werden muss. Maßnahmen des Sekretmanagements wie die Applikation eines mechanischen Insufflators-Exsufflators, die Verwendung von Trachealkanülen mit subglottischer Absaugung oder eine anticholinerge Medikation können erforderlich sein.

Die Entwöhnung von der Trachealkanüle beginnt schon während der Beatmungsentwöhnung. Je kooperativer der Patient ist, desto aussichtsreicher sind atmungstherapeutische und logopädische Behandlungsansätze. Vor dem Hintergrund der klinischen Situation neurologischer Patienten ist die interdisziplinäre Behandlung unter Einsatz von 300 Therapieminuten schon während der Beatmungsentwöhnung wichtig. Die Strategie, zunächst die Beatmungsentwöhnung durchzuführen und anschließend erst die NNFR, ist weder medizinisch-inhaltlich vertretbar noch kosteneffizient.

## Das Zertifikat „Zentrum für Beatmungsentwöhnung in der neurologisch-neurochirurgischen Frührehabilitation“

Die DGNR entwickelt und aktualisiert regelmäßig die Inhalte des Zertifizierungsverfahrens, um die Implementierung wissenschaftlicher und medizinischer Fortschritte im Sinne der Qualitätssicherung zu unterstützen. Die DGNR stellt des Weiteren den Zertifizierungsausschuss (ZA), der über die Vergabe des Zertifikats entscheidet, und benennt Fachexperten als Fachauditoren.

**Figure 2:**
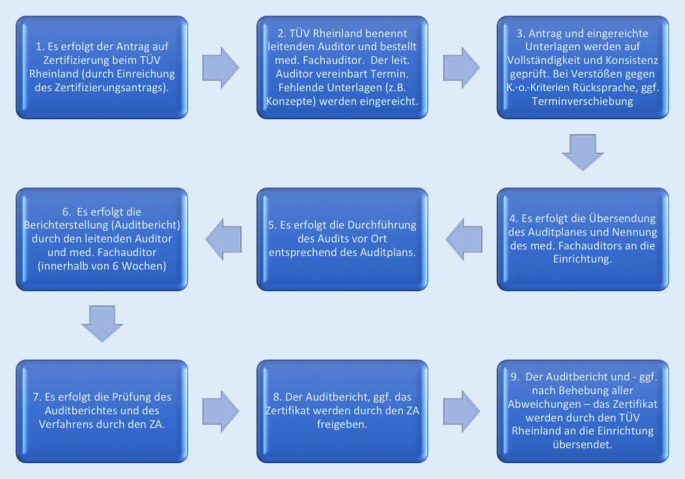

Der Leiter eines Beatmungsentwöhnungszentrums stellt den Antrag auf Zertifizierung an den TÜV Rheinland durch Einreichung des vollständig ausgefüllten Erhebungsbogens, der auf der Homepage der DGNR heruntergeladen werden kann. Die an einem Tag stattfindende Vorortbegehung sowie die Beurteilung erfolgt durch den leitenden Auditor, gestellt durch den TÜV Rheinland, und den Fachauditor. Beim Audit finden die Besprechung des Erhebungsbogens, eine Besichtigung der Beatmungsentwöhnungs- und NNFR-Stationen sowie die Überprüfung stichprobenartig gezogener Patientenakten statt. Das Gutachten der Auditoren wird den Mitgliedern des ZA übermittelt, die es kommentieren und freigegeben. Danach wird es vom TÜV Rheinland an den Antragsteller übersandt, direkt mit der Zertifizierungsurkunde, wenn keine Abweichungen vorliegen, ansonsten mit einem Katalog der noch zu erfüllenden Kriterien. Abb. 2 zeigt die einzelnen Schritte des Zertifizierungsprozesses in der Übersicht.

Die Einrichtungen der NNFR, die eine Entwöhnung von der Beatmung durchführen, sind organisatorisch heterogen. Einzelne Organisationseinheiten innerhalb einer Einrichtung werden z. B. als „Fachabteilung“, „Abteilung“, „Klinik“ oder „Universitätsklinik“ bezeichnet. Es gibt auch Frührehabilitationseinrichtungen, die prolongierte Beatmungsentwöhnung durchführen und Fachkliniken sind – auch als sog. gemischte Anstalten mit einem Krankenhausanteil mit Intensivbereich und einem Anteil als Rehabilitationseinrichtung. Die Zertifizierung ist nicht an eine bestimmte Organisationform gebunden. Auch spielt es keine Rolle, ob die Beatmungsentwöhnungseinheit als (Teil einer) Intensivstation oder Station der Frührehabilitation benannt ist. Wichtig hingegen ist, dass die im Rahmen der Zertifizierung operationalisierten Struktur- und Prozesskriterien nachweislich umgesetzt sind.

Nicht alle erforderlichen Ressourcen müssen in einem Zentrum selbst vorgehalten werden, teilweise können sie am Standort oder in unmittelbarer Nähe des Standorts vorhanden sein. Bei der Computertomographie oder der Magnetresonanztomographie ist es für die Erlangung des Zertifikats ausreichend, wenn sie zeitnah verfügbar sind.

## Vergleich mit den Zertifizierungskriterien der „Weaningzentren“ (DGP) und des Zertifikats „Entwöhnung von der Beatmung“ (DGAI)

Die Abfragen der Strukturkriterien durch die Fachgesellschaften DGP, DGAI und DGNR sind ähnlich aufgebaut, es gibt aber wichtige inhaltliche Unterschiede.

Die ärztliche Leitung soll bei den Zentren der DGP durch einen Pneumologen erfolgen, bei denen der DGAI durch einen Anästhesiologen mit Zusatzbezeichnung Intensivmedizin. Bei der Zertifizierung durch die DGNR sind bezüglich der Leitungsstruktur sowohl die Kriterien der NNFR entsprechend des OPS 8‑552, als auch die Kriterien für die prolongierte Beatmungsentwöhnung auf spezialisierter Beatmungsentwöhnungs-Einheit gemäß OPS 8-718.8 oder 8-718.9 zu erfüllen. Die Möglichkeit kooperativer, fachbereichsübergreifender Leitungsmodelle wurde berücksichtigt, da es sich bei der Beatmungsmedizin in der NNFR um ein interdisziplinäres Fach handelt.

Zentren der DGAI müssen 20 Fälle der Beatmungsentwöhnung der Gruppe 3 pro Kalenderjahr vorweisen, um zertifiziert werden zu können, während die DGP 40 Fälle fordert. Die Zentren der DGNR müssen ebenfalls die jährliche Mindestzahl von 40 Fällen behandeln, um fundierte Erfahrung in der Methode nachzuweisen.

Im Unterschied zu den Zertifizierungen der DGAI und der DGP werden durch die DGNR Strukturen und Prozessmerkmale gefordert, wie sie im OPS 8‑552 „Neurologisch-neurochirurgische Frührehabilitation“ beschrieben sind. Dies berücksichtigt, dass schon während der Beatmungsentwöhnung, aber auch danach eine Frührehabilitation erforderlich ist.

Während die DGP eine eigene Station für außerklinische Beatmung fordert, ist dies in den Zertifizierungskonzepten der DGAI und der DGNR nicht vorgesehen. Aufgrund der wachsenden Bedeutung der außerklinischen Beatmung, wird im Erhebungsbogen der DGNR die Zahl von Einstellungen auf eine außerklinische Beatmung verpflichtend abgefragt.

Die Zertifizierung der DGP fordert die Beschäftigung von Atmungstherapeuten am Zentrum, die der DGAI jedoch nicht. Die ca. zweijährige Weiterbildung zum Atmungstherapeuten wird von der DGP und der Deutschen Gesellschaft für pflegerische Weiterbildung (DGpW) angeboten. In der Weiterbildung erwerben Pflegekräfte oder Therapeuten umfangreiche beatmungsmedizinische Kenntnisse, sodass sie delegierte ärztliche Tätigkeiten übernehmen und als Multiplikatoren Teams in der Beatmungsmedizin weiterbilden können. Die DGNR erfasst im Erhebungsbogen den Einsatz dieses noch relativ neuen Berufsbildes.

Nur in den Zentren, die durch die DGNR zertifiziert werden, wird die Schluckdiagnostik mittels der fiberendoskopischen Evaluation des Schluckens (FEES) gefordert. Da viele neurologische Patienten eine neurogene Dysphagie aufweisen, kommt der Beurteilung des Schluckens und des Speichelmanagements bei der Beatmungs- und Trachealkanülenentwöhnung besondere Bedeutung zu.

Die DGAI verfügt nicht über ein Beatmungsentwöhnungsregister. Bei der DGP wiederum ist die Teilnahme am Beatmungsentwöhnungsregister für den Erhalt der Zertifizierung notwendig. Das DGNR-Präsidium prüft aktuell die Einrichtung eines Beatmungsentwöhnungsregisters.

## Fazit

Die Zertifizierung von Zentren für Beatmungsentwöhnung in der neurologisch-neurochirurgischen Frührehabilitation durch die DGNR dient dem Nachweis der Spezialisierung auf die Beatmungsentwöhnung bei neurologischen Patienten. Objektive Struktur- und Prozesskriterien, die für eine adäquate Versorgung dieser Klientel für erforderlich gehalten werden, werden durch das Zertifikat erfasst und damit wird ein qualitativ hochwertiger Behandlungsansatz dokumentiert. Die Rolle der Neurologie in der Beatmungsmedizin in Deutschland wird durch die Zertifizierung beschrieben. Auch kann so der Dialog mit anderen beatmungsmedizinischen Disziplinen, die Diskussion mit Politikern und Kostenträgern und die Kommunikation mit den Betroffenenverbänden zukünftig erleichtert werden. Damit ist die Zertifizierung eine wichtige Grundlage für einen konstruktiven Austausch über Behandlungsinhalte und -kapazitäten.
